# Association of Ischemic Cardiovascular Disease with Inadequacy of Liver Store of Retinol in Elderly Individuals

**DOI:** 10.1155/2018/9785231

**Published:** 2018-04-02

**Authors:** I. O. L. Lima, W. A. F. Peres, S. Cruz, A. Ramalho

**Affiliations:** ^1^Federal University of Rio de Janeiro, Rio de Janeiro, RJ, Brazil; ^2^NPqM/UFRJ, Rio de Janeiro, RJ, Brazil

## Abstract

**Objective:**

Vitamin A antioxidant role has an important relationship with the metabolic processes of aging and cardiovascular disease (CVD). This study aimed at assessing the liver store of retinol in elderly individuals who died from cardiovascular disease and its relationship with liver weight and body weight.

**Methods and Results:**

This is a cross-sectional study conducted in necropsied individuals, aged 60 years or over, until 48 hours postmortem. The study assessed 65 elderly individuals who died from ischemic heart diseases (G1), cerebrovascular diseases (G2), other forms of heart disease (G3), or infectious heart diseases (G4). Twenty percent had inadequate liver store of retinol. G1 showed lower median of liver store of retinol when compared to G3 (*p* < 0.001), and G3 showed the highest median when compared to G2 (*p* = 0.007). A significant association was observed between inadequate liver store of retinol and death by ischemic CVD (G1) (*p* = 0.001) with an odds ratio of 10.38. It was observed that individuals with higher body weight and liver weight showed lower liver store of retinol with significant differences (*p* = 0.027 and *p* = 0.026).

**Conclusion:**

Ischemic CVD and increased body weight and liver weight are related to a greater impairment of the liver store of retinol.

## 1. Introduction

The process of population ageing and its epidemiological consequences, such as an increasing mortality rate due to chronic noncommunicable diseases (NCDs), have been highlighted worldwide. Epidemiological studies [1, 2] indicate that the main causes of death in the elderly population, especially the young elderly (60–69 years), are due to cardiovascular diseases (CVDs), mainly those of ischemic origin [3].

In Brazil, there has been a gradual increase in the number of deaths from ischemic heart diseases (IHDs) totaling 77,162 deaths in 2011. IHDs are associated with damage to the blood supply of coronary arteries caused by blockage of these vessels by atherosclerotic plaques that stimulate the circulation and aggregation of proinflammatory molecules, favoring increased local oxidative stress and organic antioxidant activity [4]. Thus, it is suggested that ischemic diseases demand a larger amount of antioxidants for their prevention and treatment, when compared to other nonischemic CVDs.

Vitamin A is an important fat-soluble micronutrient that has a wide range of physiological functions. Hepatic stellate cells (HSCs) are major storage sites for vitamin A. During liver injury, stellate cells activate into alpha smooth muscle actin-expressing contractile myofibroblasts and lose their retinol content, subsequently leading to liver fibrosis [5]. Vitamin A and its derivate retinoids exhibit potent antiproliferative and anti-inflammatory activities by their ability to regulate gene expression in target cells by binding to nuclear receptors that are ligand-dependent transcription factors, known as retinoic acid receptors and retinoid X receptors [6]. The role of vitamin A and its metabolites in the immune system potentiates the actions of T-cell proliferation, B-cell activation, T helper cell (TH1 and TH2) balance, and differentiation of regulatory T-cells (Treg cells) [7]. Also, vitamin A status contributes to the regulation of carbohydrate metabolism in the liver by means of regulation of hepatic glycogen metabolism, glycolysis, and gluconeogenesis. Regarding the role of vitamin A status in lipid metabolism, it has been demonstrated in experimental studies that both hypervitaminosis A and vitamin A deficiency (VAD) can contribute to triglyceride accumulation in the liver or to fatty liver by increasing hepatic lipid synthesis, although by different mechanisms [8].

The increased demand of antioxidant by ischemic CVDs creates a greater dependency level of exogenous antioxidants for control of oxidative stress. Among these antioxidants is vitamin A, whose role consists in delaying or preventing the initiation of atherosclerotic processes, such as peroxidation, in the cellular lipid component and the generation of hydroperoxides [9].

The atherosclerotic process has also a relationship with other inflammation-related chronic diseases such as obesity which is currently recognized as a multifactorial disease and is found in 17.9% of the Brazilian population [10]. Excess weight has also a greater antioxidant demand since there is a higher prevalence of VAD in obese patients. In fact, vitamin A plays an important role in the expansion and maturation of the adipose tissue during obesity [11]. Also, nonalcoholic fatty liver disease (NAFLD) is frequently associated with atherosclerosis [12].

Among the methods used for assessing the nutritional status of vitamin A, the liver store of retinol is considered the gold standard method since the liver has been acknowledged as the main storage location of vitamin A and since serum concentration of retinol does not directly reflect the liver store of this nutrient [13].

Therefore, our assumption is that individuals who died from CVD of ischemic etiology show a greater impairment of the liver store of retinol, besides indicating a significant association between the liver store of retinol, body weight, and liver weight.

## 2. Methods

This is a cross-sectional analytical study carried out with database of individuals necropsied in the Legal Medical Institute *(Instituto Médico Legal (IML))* in the Municipality of Rio de Janeiro from 2010 to 2012.

The study comprised 65 individuals of both genders, regardless of ethnicity, aged 60 years or over, who died from CVD, and necropsied until 48 hours postmortem.

Information was collected on age, gender, body weight, liver weight, and the type of CVD that caused death. The cause of death recorded by the coroner on the death certificate was considered its root cause. The next stage was to group the causes of death from CVD according to the International Classification of Diseases, 10th Revision (CID-10) [14], and classify those groups according to the causes presented.

Liver sample collection was performed by properly trained IML technicians in individuals undergoing autopsy, in 3 weekly shifts of a 06-hour shift, on alternate weekdays, including 1 (one) day of the weekend and a night shift, and in two of the IML/RJ necropsy procedure rooms, chosen randomly at every shift. During the autopsy, through the opening of the abdominal cavity and using an autopsy knife and Mayo scissors, the liver was exposed and, subsequently, a sample of approximately 2 g was removed from it. Samples were identified and stored in a freezer at a temperature equal to or less than 20°C until liver retinol quantification.

For quantification, liver samples were homogenized with a 50% glycerol solution in distilled water and processed in accordance with Flores and coworkers' recommendations [15]. Determination of the liver concentration of retinol was performed by the method of high-performance liquid chromatography with ultraviolet detector (HPLC-UV) in accordance with the technique described by Hess and coworkers [16].

In accordance with the proposal of Olson and coworkers [17], concentrations of liver retinol (LR) were quantified as unesterified retinol and classified as adequate when values were higher than or equal to 20 *μ*g/g and inadequate when values were lower than 20 *μ*g/g.

Variable distribution was identified as nonnormal by the Kolgomorov-Smirnov test. Descriptive analysis of variables was performed such as measure of central tendency and dispersion. Multiple comparisons of numeric variables between groups of causes of death were performed by the Kruskal-Wallis variance analysis. Once a significant difference was identified between medians of liver retinol in the groups, at 5%, the Mann-Whitney nonparametric test was applied to each pair of groups separately. We considered a level of 0.8% (5% divided by the number of possible classifications) in order to control type 1 errors (*α* errors), which imply mistakenly finding significant differences. We used the Mann-Whitney test to compare numerical variables between the two groups. Correlations between numerical variables were tested using the Spearman coefficient. The association between categorical variables was performed by the chi-square test (*χ*^2^), and the odds ratio was also calculated. Linear, bivariate, and multivariate regressions were performed. Analyses were carried out in the Statistical Package for the Social Sciences (SPSS) for Windows, version 21.0. A 5% (*p* < 0.05) significance level was adopted.

The current study was approved by the Ethics Committee of Rio de Janeiro IML (number 0351.2713.000.555). Since it assessed deaths that needed investigation to establish their causes, this study was inserted in the category of cases in which no informed consent (IC) is required.

## 3. Results

A total of 65 elderly patients were included in the study, and 34 were male (52.3%). Median age was 64 years (60–85), and 90.8% of the sample was in the 60- to 69-year age group (Table 1).

Types of common causes of death from CVD were classified in the following groups: G1—ischemic heart diseases (CID-10 20-I25); G2—cerebrovascular diseases (CID-10: I60-I69); G3—other forms of heart disease (such as noninfectious rheumatic diseases, heart diseases, heart failure, and hypertension) (CID-10: I05.0-I08.9 and I10-I15.9 and I24 and I31.2-I31.9 and I34-I37.9 and I39-I39.4 and I42-I51.9); and G4—heart infectious diseases (such as endocarditis, myocarditis, pericarditis, and derived or not from rheumatic disease) (CID: I09.0-I09.9 and I30.0-I30.9 and I30.0-I31.1 and I32.0-I33.9 and I38 and I39.8-I41.8).

It was observed that 44.6% (*n* = 29) of the elderly individuals died from ischemic heart diseases, 29.2% (*n* = 19) from cerebrovascular diseases, 18.5% (*n* = 12) from other forms of heart disease, and 7.7% from infectious heart diseases.

In total, men showed lower median of liver retinol (*p* = 0.016) when compared to women. In assessing the correlation between body weight and liver weight, a significant and positive correlation was observed between body weight and liver weight (*r* = 0.852 com *p* < 0.001).

As regards the liver store of vitamin A, 20% of the sample showed inadequacy of storage, 100% (*n* = 13) was in the 60- to 69-year age group, and 84.6% (*n* = 11) was men and died from ischemic diseases. No individual in G3 and G4 showed inadequate liver store of retinol (Table 1).

No significant differences were found between age and gender group in the groups whose causes of death were CVD (*p* = 0.389 and *p* = 0.06, resp.), which suggests that the main factor for decrease or increase in the liver store of retinol, liver weight, or body weight is more related to the etiology of the associated disease.

In the analysis of multiple comparisons, a significant difference was found between the medians of liver retinol in the groups (*p* < 0.001). In this analysis of each pair of groups separately, G1 showed the lowest median when compared to G3 (*p* < 0.001), and G3 showed the highest median when compared to G2 (*p* = 0.007) (Table 2).

In addition, both in the linear and bivariate logistic regressions, the inadequacy of liver retinol was higher for ischemia (*p* = 0.002) and myocardiopathies (*p* = 0.002) when compared to other groups. Thus, one can propose that both causes of death can reduce serum concentrations of liver retinol, with 19.6% and 26.1% chances, respectively. And yet, the multivariate regression confirms that the causes of deaths resulting from ischemia (*p* = 0.045) and cardiomyopathies (*p* = 0.028) are related to inadequacy of retinol (*p* = 0.001).

A significant association was observed between inadequate liver store of retinol and death by ischemic CVD (*p* = 0.001), with an odds ratio of 10.38 for inadequacy of store in ischemic heart disease.

In the sample studied, it was observed that individuals with higher body weight and liver weight showed lower liver store of retinol with significant differences (*p* = 0.027 and *p* = 0.026).

## 4. Discussion

In the current study, the liver store of retinol was assessed in the liver tissue of the elderly individuals who died from CVD. Those whose deaths resulted from ischemic heart disease showed an increased presence of VAD with significantly lower concentration of vitamin A stored in the liver when compared to other causes of death, and mostly were males. To our knowledge, this is the first study that assesses vitamin A liver store of retinol in the elderly who died from CVD.

A high prevalence of VAD was observed in the elderly who died from CVD, a finding that may be regarded as a serious public health problem, considering that the World Health Organization (WHO) establishes as cut-off point for VAD a prevalence above 20% [18].

In Brazil, ischemic heart diseases are the leading cause of death in the elderly aged 65–69 years, while cerebrovascular diseases are more frequent in the elderly aged 80 years and over, with increased frequency of males, which corroborates the findings of our study [19]. A study on the prevalence of inadequate micronutrient intake among Brazilian adults has reported that the inadequacy of micronutrient intake in Brazil was extremely high, with vitamin A showing 78% of inadequacy in all regions studied, both in urban and rural areas [20]. These data become even more disturbing when inadequacy of consumption is acknowledged to be the main etiological factor related to VAD development.

Despite the scarcity of studies on the liver store of retinol in necropsied individuals, in a classic study conducted in Canada in 1968, in a sample with individuals who died from various causes, it was observed that those who died from CVD showed lower concentrations of liver vitamin A when compared to other diseases, with median of 111 *μ*g/g of liver [21]. In our study, the median of vitamin A stored in the liver of all patients was 101.3 *μ*g/g of liver, corroborating the finding of the aforementioned study [21].

In another study addressing individuals who died from various causes in the United States of America (USA), it was observed that 21% of patients with CVD had VAD [22]. Also, a prospective cohort study reported that elderly individuals (>50 years) have a higher risk of death from CVD in case of deficiency and/or excess of vitamin A (≤30 mg/dL or ≥80 mg/dL) [23], corroborating the data of our study. However, a study conducted in China showed that the reduced concentrations of retinol in individuals aged 43 to 74 years do not have a relationship with acute myocardial infarction [24].

It has been highlighted that the reduced serum concentrations of retinol (<601 g/l) in middle-aged men (≥50 years) in an isolated way have risks similar to the classic risk factors such as HDL cholesterol and interleukin-6 concentrations for occurrence of coronary cardiovascular diseases [25].

In a more recent study, Ramalho and coworkers [26] reported that adult individuals who died from CVD showed higher liver store depletion when compared to other causes of death, and among those who died from CVD a total of 38.0% showed inadequacy of liver store of vitamin A and died from CVD of ischemic etiology. In addition, the authors observed that, in general, the individuals more affected by CVD were adults in the highest age group. These data corroborate the findings of the current study and support the assumption that the aging process may cause a more expressive loss of retinol store in CVDs.

This new perspective found in senescent populations has been supported by extensive literature that reported a greater demand of antioxidant nutrients in the development and treatment of CVD of ischemic etiology [27], in addition to the progressive decrease in the storage capacity of vitamin A that occurs with age [28]. The study of Sundboom and Olson [28] has shown that the deposition of retinyl ester in micellar form in the liver was inversely related to age in rats, which corroborates the results found in the current study.

Besides the changes related to damage to vitamin A storage, it must be considered that factors related to heart aging such as functional, structural, and cellular changes, both on vascular and cardiac levels, and reduction of superoxide dismutase and nitric oxide bioavailability jointly contribute to increased oxidative stress in senescence, which in the long run may be related to a lower liver store of vitamin A as demand increases [29].

The relationship between the greatest demand for vitamin A and the increase in oxidative stress, which was shown during ischemic CVD, was reported by studies that have found an association between low serum concentration of vitamin A and diagnosis of symptomatic and asymptomatic carotid atheroscleroses [30, 31]. These results, supported by the significant action of vitamin A in the reduction of lipid peroxidation [9], may explain the lower medians of liver retinol found in G1 and G2, considering that nearly 85% of cerebrovascular accidents (CVA) are of ischemic etiology [32], with no significant difference in the liver store of vitamin A between these two groups.

The relationship between liver store of retinol and body weight found in our study is in line with the finding of Bento [33], who reported a significant reduction of the serum concentrations of retinol with an increase in body weight and body mass index, showing VAD as an aggravating factor for increase in body adiposity. The role of vitamin A in the regulation of the inflammatory response in low-grade inflammation diseases, such as obesity, has been well established. VAD has a relationship with inadequacy of Th1 and Th2 lymphocyte responses and is associated with an increased expression of leptin, causing increased and decreased production of pro- and anti-inflammatory cytokines, respectively [34].

According to a recent systematic review (2017), there is a greater risk of cardiovascular mortality and morbidity associated with obesity in that the BMI may be associated with high blood pressure (hypertension), considered a main risk factor for its development. Thus, both obesity and hypertension can facilitate vascular dysfunction, involved with oxidative stress and inflammatory signals [35].

In addition, weight gain, which is often pointed out in CVDs, has a well-established relationship with a stimulus to accumulate visceral fat related to hepatic steatosis [36]. Although liver fat has not been assessed in our study, it may be inferred that fatty liver disease, a common comorbidity in CVD, could be present in these individuals and be related to increased body weight and liver weight. Considering this panorama, similar results were found by Chaves and coworkers [37], who reported a prevalence of 67.5% of inadequacy of liver store of retinol in obese subjects with nonalcoholic fatty liver disease (NAFLD), in addition to reporting a negative association between severity of histological NAFLD and concentration of liver retinol. Moreover, the patatin-like phospholipase domain-containing protein 3 (PNPLA3) gene is involved in retinol metabolism in HSCs since PNPLA3 hydrolyzes retinyl palmitate in human hepatic stellate cells (HSC) [38]. It has been demonstrated that this enzyme activity is markedly reduced in the presence of a single nucleotide polymorphism (SNP) of the patatin-like phospholipase domain-containing 3 gene (PNPLA3) that results in an isoleucine to methionine substitution at position 148 (I148M) [39]. This *PNPLA3* I148M variant has been shown to be associated with more severe diseases on the spectrum of NAFLD patients. A recent study suggests that NAFLD is related to subclinical atherosclerosis when it is more associated with the metabolic disorders than to the genetic variation [40].

The current study has some limitations since it has not considered possible liver damages, given the exclusive use of the root cause of death in its design. Considering the postmortem events, the cadaveric body edema can compromise the use of body weight in the assessment of nutritional status, especially regarding individuals with heart disease. However, studies report that the liver, unlike what is observed in other organs, shows no weight change after death in the period covered by this study. In addition, the positive correlations found in the literature between liver weight and body weight, height, and body mass index [41, 42] value the use of these associated markers, corroborating the finding of the current study regarding the correlation between the body weight and liver weight.

## 5. Conclusion

Given the findings presented here, we conclude that CVD of ischemic etiology and the increase in body weight and liver weight are related to the greater impairment of the liver store of retinol in the elderly population, mainly in elderly men aged 60–69 years. Emphasizing the originality of the work and the fact that the elderly population is not considered a risk group for VAD development, the results of our study call attention to the adequacy of the nutritional status of vitamin A in individuals with CVD and present themselves as elements to be used as a supporting strategy for the treatment of cardiovascular diseases.

## Figures and Tables

**Table 1:** Frequency of distribution of demographic, clinical, and biochemical variables in frequency, medians, and minimum and maximum values of the total sample and of the sample with inadequacy of liver retinol.

Variables	Frequency (*n* = 65) % (*n*)	Sample with inadequacy of liver retinol Frequency (*n* = 13) % (*n*)
*Age*		
60–69 years	90.8 (59)	100 (13)
>70 years	9.2 (06)	—
*Gender*		
Male	52.3 (34)	84.6 (11)
Female	47.7 (31)	15.4 (02)
*Causes of death*		
G1	44.6 (29)	84.6 (11)
G2	29.2 (19)	15.4 (02)
G3	18.5 (12)	
G4	7.7 (05)	
*Liver retinol*		
Adequate	80.0 (52)	
Inadequate	20.0 (13)	
Variables	Median (minimum–maximum)	Median (minimum–maximum)
Age (years)	64 (60–85)	64 (60–67)
Liver retinol (*μ*g)	101.3 (7.0–366.9)	12.2 (7.0–18.5)
Body weight (kg)	65 (23.3–96.6)	75 (46–96.6)
Liver weight (g)	1500 (1050–2500)	1940 (1150–2500)

G1: ischemic heart disease; G2: cerebrovascular diseases; G3: other heart diseases; G4: infectious heart diseases.

**Table 2:** Comparisons of liver store of retinol according to causes of death from CVD.

Groups	Median (*μ*g)	Interquartile range	*p* value
G1 × G2	56.06–128.99	111.79–198.98	0.012
G1 × G3	56.06–294.99	111.79–140.3	0.001^∗^
G1 × G4	56.06–128.99	111.79–166.73	0.134
G2 × G3	128.99–294.99	140.3–198.98	0.007^∗^
G2 × G4	128.99–128.99	166.73–198.98	0.749
G3 × G4	294.99–128.99	140.3–166.73	0.017

Mann–Whitney test, ^∗^significant comparisons *p* < 0.008, G1: ischemic heart disease; G2: cerebrovascular diseases; G3: other heart diseases; G4: infectious heart diseases; LR: liver retinol.
